# The Association between Energy-Adjusted Dietary Inflammatory Index, Body Composition, and Anthropometric Indices in COVID-19-Infected Patients: A Case-Control Study in Shiraz, Iran

**DOI:** 10.1155/2022/5452488

**Published:** 2022-03-29

**Authors:** Donya Firoozi, Seyed Jalil Masoumi, Sara Ranjbar, Nitin Shivappa, James R. Hebert, Morteza Zare, Hossein Poustchi, Faeze Sadat Hoseini

**Affiliations:** ^1^Student Research Committee, School of Nutrition and Food Sciences, Shiraz University of Medical Sciences, Shiraz, Iran; ^2^Nutrition Research Center, School of Nutrition and Food Sciences, Shiraz University of Medical Science, Shiraz, Iran; ^3^Center for Cohort Study of SUMS Employees' Health, Shiraz University of Medical Sciences, Shiraz, Iran; ^4^Gastroenterohepatology Research Center, Shiraz University of Medical Sciences, Shiraz, Iran; ^5^Cancer Prevention and Control Program and Department of Epidemiology and Biostatistics, Arnold School of Public Health, University of South Carolina, Columbia, SC, USA; ^6^Department of Nutrition, Connecting Health Innovations LLC, Columbia, SC, USA; ^7^Digestive Oncology Research Center, Digestive Diseases Research Institute, Shariati Hospital, Tehran University of Medical Sciences, Tehran, Iran; ^8^Digestive Diseases Research Center, Digestive Diseases Research Institute, Shariati Hospital, Tehran University of Medical Sciences, Tehran, Iran; ^9^Liver and Pancreatobiliary Diseases Research Center, Digestive Diseases Research Institute, Shariati Hospital, Tehran University of Medical Sciences, Tehran, Iran; ^10^Department of Nutrition, Science and Research Branch, Islamic Azad University, Tehran, Iran

## Abstract

**Background and Aims:**

Inflammation is strongly associated with the severity and mortality rate of SARS-CoV-2 disease (COVID-19). Dietary factors have a crucial role in preventing chronic and systemic inflammation. This study aimed to evaluate the association between energy-adjusted dietary inflammatory index (E-DII) scores and body composition parameters in COVID-19-infected patients compared to noninfected controls.

**Methods:**

A total of 133 COVID-19-infected patients and 322 noninfected controls were selected and enrolled from the Cohort Study of Employees of Shiraz University of Medical Sciences. E-DII score was calculated based on a validated food frequency questionnaire (FFQ) and body composition was measured using In-Body 770 equipment. Logistic regression models were utilized to estimate the odds ratio (OR).

**Results:**

In the control group, the mean E-DII score was significantly lower than the case group (−2.05 vs. −0.30, *P* ≤ 0.001), indicating that the diet of COVID-19-infected subjects was more proinflammatory than the controls. For every 1 unit increase in E-DII score, the odds of infection with COVID-19 was nearly triple (OR: 2.86, CI: 2.30, 3.35, *P* ≤ 0.001). Moreover, for each unit increase in body mass index (BMI), the odds of infection to COVID-19 increased by 7% (OR: 1.07, CI: 1.01, 1.13, *P* = 0.02). No significant difference was observed for other anthropometric parameters.

**Conclusion:**

The findings revealed that obese people and those consuming a more proinflammatory diet were more susceptible to coronavirus infection. Therefore, maintaining ideal body weight and consuming a more anti-inflammatory diet can decrease the probability of COVID-19 infection.

## 1. Introduction

Coronavirus disease (COVID-19) was first detected with apparent pneumonia in Wuhan, China, in December 2019. After about 1.5 years, the disease became pandemic and was confirmed by the World Health Organization (WHO) in March 2020 as a major threat to global public health [1]. According to the latest WHO news, more than 267.86 million confirmed cases and 5.28 million deaths from COVID-19 have been reported by 10 December 2021 [2]. Given the high prevalence and the absence of a definite way to eradicate the disease, scientists have looked for a way to reduce mortality and prevent infection [3]. COVID-19 patients with elevated inflammatory cytokines were shown to be more at the mortality risk [4].

The immune system is an important flexible factor in preventing and controlling respiratory viral infections and mortality [5]. A weak immune system as a risk factor is associated with disease severity and worse outcomes in people with viral infections such as influenza, adenovirus, and COVID-19 [5]. In COVID-19 patients, severe inflammation due to cytokine storm and impaired immune function leads to multiple organ failure. Defect in the immune system is a risk factor for COVID-19 and inflammation is a major cause [6, 7] of death, so strengthening the immune system and dealing with inflammatory factors may reduce the incidence and mortality of this disease [5].

Nutrition and optimal diet play a crucial role in strengthening the immune system and also in reducing inflammation and lung tissue damage [8, 9]. Indeed, several dietary factors were demonstrated to influence inflammation. More specifically, dietary patterns with low saturated and trans-fatty acids intake and high consumption of high omega-3, fruits, vegetables, nuts, and whole grains have been associated with a lower level of inflammation [10,11]. To determine the correlation between diet and inflammation, the dietary inflammatory index (DII®) was developed that can evaluate the inflammatory potential of a diet from maximally anti-inflammatory to maximally proinflammatory levels [12]. The E-DII score has been verified by such inflammatory markers, like tumor necrosis factor alpha (TNF*α*), interleukin-1 (IL-1) and interleukin-2 (IL-2), and C-reactive protein (CRP) [5].

Previous studies demonstrated that higher DII was associated with impaired lung function [13]. Moreover, subjects with asthma or wheeze consumed a more proinflammatory diet [14]. Furthermore, by increasing production of inflammatory mediators obesity leads to inflammation in the airways, which is directly related to asthma [14].

Our hypothesis was that subjects with COVID-19 infection have higher DII and and BMI, indicating the potential of a proinflammatory diet, than those of noninfectious subjects. The aim of this study was to investigate the association between energy-adjusted dietary inflammatory index (E-DII), body composition, and anthropometric parameters with COVID-19 in a case-control study in southern Iran. Considering (i) the high prevalence and mortality of COVID-19 and its relationship to the inflammatory state of the body and (ii) the impact of diet on the immune system and inflammatory status, the focused clinical question we aim to address is whether the updated dietary approaches would help reduce the risk of COVID-19 and its associated inflammation?

## 2. Materials and Methods

### 2.1. Participants

This is a retrospective case-control study on participants nested within the Cohort Study of Shiraz University of Medical Sciences (SUMS) for Employee Health in southern Iran (SUMS EHCS, a branch of PERSIAN Cohort Study). From March 2020 to June 2020 in SUMS EHCS, a total of 3000 male and female participants aged 20–70 years were screened for a probable COVID-19 infection, while 133 cases were diagnosed as COVID-19-infected patients. COVID-19 was assessed by quantitative real-time polymerase chain reaction (qPCR). Moreover, some of the participants had the results of chest computed tomography scans and serologic tests. The control group was randomly selected from 3,000 participants who were not infected to COVID-19 verified by qPCR. To optimize power, approximately 2.5 controls were included for each case (*n* = 332). All participants provided written informed consent and the study was approved by the Ethics Committee of Shiraz University of Medical Sciences (Code: IR.SUMS.REC.1396.S441), undertaking ethical principles for medical research involving human subjects of the World Medical Association Declaration of Helsinki.

### 2.2. Inclusion and Exclusion Criteria

Individuals were excluded from the study if data on body composition, dietary intake, and demographic information were incomplete or if they were pregnant, breastfeeding, or on a specific diet at the time of data collection.

### 2.3. Assessment of General Characteristics of Participants

Trained interviewers were used in the study who utilized a general questionnaire to complete demographic information of participants such as age, sex, ethnicity, level of education (under diploma, diploma degree, postdiploma, bachelor's degree, master's, and Ph.D. degree), marital status, and smoking habits.

### 2.4. Assessment of Dietary Intake

To assess dietary intake, participants were asked to respond to a 116-item semiquantitative food frequency questionnaire (FFQ). The reliability and validity of the FFQ have been confirmed in a previous study [15]. Participants were requested to report the amount and frequency of consumption of the food items in the last year, on a daily, weekly, monthly, and yearly basis. All information was collected from patients through face-to-face interviews. The United States Department of Agriculture (USDA) nutrient database was used to compute nutrient scores from consumed foods. Nutrient scores were derived applying the Nutritionist IV diet analysis software (version 7.0; Nsquared computing, Salem, OR, USA) to determine energy and gram of nutrient intake.

### 2.5. Assessment of Anthropometric and Body Composition

Body composition parameters, including weight, body mass index (BMI = weight (kg)/height (m) 2), fat-free mass (FFM), skeletal muscle mass (SMM), body fat mass (BFM), and visceral fat area (VFA), were determined using In-Body 770 equipment. Also, height (in cm), weight (in kg), waist, hip, and waist circumferences (in cm) were measured based on US National Institutes of Health protocols [16]. The waist-to-hip ratio (WHR) was calculated by dividing the waist circumference (cm) to hip circumference (cm). In order to lessen the error, all measurements were taken in the morning, when participants were fasting and emptied the bladder.

### 2.6. Assessment of E-DII

The DII® is based upon up to 45 food parameters, which have been scored based on reported proinflammatory or anti-inflammatory effects on specific inflammatory markers (IL-1*β*, IL-4, IL-6, IL-10, TNF-*α*, and CRP) using 1943 peer-reviewed articles published until December 2010. Details of the development and initial testing of the DII® have been described before [17]. Over 30 construct validation studies are available all over the world using a variety of inflammatory biomarkers. Briefly, the scoring algorithm uses a global reference database (food consumption from eleven populations globally) and food parameter-specific inflammatory effect scores to create an overall DII® score for an individual. The DII® scored individuals' diets on a continuum from strongly anti-inflammatory (−8.87) to strongly proinflammatory (+7.98).

To calculate DII® scores for the participants, dietary intake data were used to calculate an individual's intake of food parameters and were compared to the global reference database. For each participant, a Z-score for every food parameter was calculated based on the global mean and standard deviation; this was achieved by subtracting the global mean from the amount reported and dividing this value by the standard deviation. The Z-scores were converted to a proportion to minimize the effects of outliers (“right-skewing”). The standardized dietary intake data (proportion) were centered by doubling and subtracting 1 and then multiplied by the inflammatory effect score of each food parameter and summed to obtain an overall DII score for every participant in the study. A total of 29 food parameters were available from the food diaries to compute the overall DII® scores. These included energy, carbohydrate, protein, total fat, fiber, cholesterol, saturated fat, monounsaturated fat, polyunsaturated fat, omega-3 fatty acids, omega-6 fatty acids, niacin, thiamine, riboflavin, iron, magnesium, zinc, vitamin C, vitamin E, folic acid, beta-carotene, caffeine, garlic, onion, and tea. The energy-adjusted DII (E-DII™) scores were calculated by converting all food parameters to the amount of consumed food per 1000 kcal [18]. To compute the E-DII score, procedures identical to those utilized to develop the DII were used, and an energy-adjusted global comparative database was applied; as energy is in the denominator, only 28 food parameters were used in the calculation of the E-DII scores.

### 2.7. Statistical Analysis

Data analysis was performed using SPSS software (version 18, Chicago, IL, USA). The Kolmogorov–Smirnov test was used to evaluate the distribution of data. Continuous and categorical variables were expressed as mean (standard deviation) and number (percent), respectively. An independent sample *t*-test was used to analyze continuous data with normal distribution, and an independent Mann–Whitney *U* test was utilized for data with nonnormal distribution. Also, Chi-square or Fisher's exact test was applied to compare categorical variables. Normally distributed data were compared with the ANOVA test and nonnormally distributed data were compared with the Kruskal–Wallis test. Categorical variables were also compared with the Chi-square test. A logistic regression model was carried out for COVID-19 as the outcome. For predicting the odds of COVID-19 infection in the case and control groups, univariate logistic regression models were used. In addition to being fit as a continuous variable, the E-DII was categorized into quartiles. To reduce the effect of confounding factors, variables with significance level ≤0.20 were included in the multivariable regression model. Odds ratios (OR) and associated 95% confidence intervals (CI) were computed from the logistic regression model. *P* values less than 0.05 were considered statistically significant.

## 3. Results

### 3.1. Evaluation of the Continuous and Categorical Variables of Participants

The characteristics of continuous and categorical variables for cases (*n* = 133) and controls (*n* = 322) were shown in Table 1. There were no significant differences in mean age and anthropometric parameters/body composition measures, including weight, height, BMI, FFM, SMM, BFM, abdominal and hip circumferences, WHR, and VFA between the case and control groups (Table 1). Furthermore, no significant differences were observed between cases and controls in terms of marital status, sex, smoking habits, or ethnicity. Education differed significantly between cases and controls (*P* ≤ 0.001).

### 3.2. E-DII Scores in Control and Case Groups

**Figure 1:**
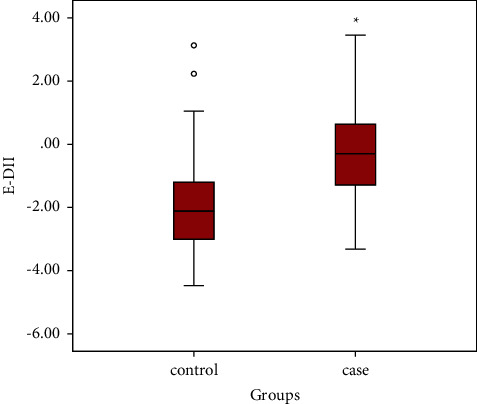
Box-plot graph of E-DII scores in control and case groups. Values are means (SE).  ^*∗*^*P* ≤ 0.001. E-DII: energy-adjusted dietary inflammatory index.

The E-DII scores ranged from −4.47, as the most anti-inflammatory score in the control group, to 3.46, as the most proinflammatory score in the case group. The mean E-DII score in the control group was significantly lower than the case group (−2.05 (1.24) vs. −0.30 (1.38), *P* ≤ 0.001), indicating that diets in COVID-19-infected patients were more proinflammatory than those of controls (Figure 1).

### 3.3. Assessment of the Characteristics of Participants According to Quartile of E-DII

When the E-DII was categorized into quartiles (Table 2), the number of cases with COVID-19 was significantly (*P* ≤ 0.001) higher in quartile 4 (E-DII ≥−0.54), while the number of controls was higher in quartile 1 (E-DII ≤−2.76). Quartile 4 contained the highest proportion of men. Also, significantly decreasing trends were observed across quartiles for more anti-inflammatory nutrients such as omega-3 fatty acid (*P* ≤ 0.001), *β*-carotene (*P* ≤ 0.001), folic acid (*P* ≤ 0.001), vitamin D (0.01), iron (*P* ≤ 0.001), magnesium (*P* ≤ 0.001), zinc (*P* = 0.002), selenium (*P* ≤ 0.001), and fiber (*P* ≤ 0.001) and significant increasing trends were noted for PUFA (*P* = 0.01), omega-6 (*P* = 0.02), and cholesterol (*P* = 0.03) (Table 2). No significant changes were seen for age, weight, BMI, smoking habits, and other nutrients across quartiles.

### 3.4. Logistic Regression Models Analysis

A multivariable logistic regression model as the outcome was fit with cases (COVID-19-infected individuals) versus controls (uninfected individuals) (Table 3). For every 1 unit increase in E-DII score, the odds of being infected with COVID-19 increased by 186% (OR: 2.86, CI: 2.30, 3.53, *P* ≤ 0.001). Also, for every 1 unit increase in BMI, the odds of getting COVID-19 increased by 7% (OR: 1.07, CI: 1.01, 1.13, *P* = 0.02). A significant odds ratio was also seen for the level of education. It was shown that an increase in the educational level resulted in decreasing the odds of getting COVID-19. The increase in the level of education from under diploma and diploma level to postdiploma and bachelor's level led to a decrease in the odds of COVID-19 infection by 85% (OR: 0.15, CI: 0.05, 0.40, *P* ≤ 0.001) and for master's and Ph.D. levels, a decrease in the odds of COVID-19 infection by 48% (OR: 0.52, CI: 0.29, 0.92, *P* = 0.02) (Table 3).

## 4. Discussion

Based on access to available data in the literature, this can be a beginning to find out the association between COVID-19 and the E-DII. In this retrospective nested case-control study, we found that participants with higher E-DII scores, indicating a diet with proinflammatory potential, were more at risk of being infected to COVID-19. Our results supported our hypothesis that consuming a proinflammatory diet is associated with an increased risk of COVID-19 infection. There was also a positive relationship between BMI and increased risk of COVID-19, and educational level was inversely correlated with the risk of COVID-19.

In previous research, a higher DII/E-DII score was shown to be correlated with a wide variety of inflammation-related diseases such as asthma [19], cancer [20], and cardiovascular diseases [21]. Unbalanced diet and malnutrition may contribute to chronic low inflammatory status, negatively impacting the immune system and making the body susceptible to disease [22]. Studies on the association between DII and respiratory diseases are rare with IL-6 and forced expiratory volume 1 (FEV1) levels, suggesting a relationship between an inflammation-inducing diet and impaired lung function [19]. Similarly, a cohort study in the general population in Australia found a negative relationship between increased DII score and lung function. In this study, IL-6 level and dietary fat intake were negative predictors of FEV1 [23].

Previous studies suggested that based on the absence of the antioxidant content, the Western diet and diet with a low amount of fruits and vegetables could increase the CRP and systemic inflammation in asthmatic patients [24]. In fact, an inflammatory diet was a predictor of systemic inflammation. Individuals who consumed a high-DII diet showed a higher level of IL-6, causing a greater risk of systemic inflammation-related diseases among them [25]. In the case of COVID-19, the production of inflammatory cytokines such as IL-6, IL-1, TNF-*α*, and CRP [25] contributed to a storm of cytokines resulting in an immune dysregulation induced by T cells and inflammatory monocytes [11].

One potential explanation for the positive association between E-DII and COVID-19 can be the role of the proinflammatory diets in the production of inflammatory mediators and cytokines, such as B and T lymphocytes, and vascular cell adhesion molecules (VCAM). Moreover, a proinflammatory diet can contribute to insulin resistance that might lead to the induction of systemic inflammation. The association between E-DII/DII scores and elevated IL-6 levels may indicate another possible mechanism associated with impaired lung function, increased CRP levels, and airway inflammatory cell counts [26]. Consistent with results from the present study, several studies that have been conducted up to now have found obesity to increase the risk of COVID-19 and its severity [27–30]. Obesity can decrease immune cells activity and cause intestinal microbiome imbalance, thus triggering inflammatory cytokine phenotype and intensifying antiviral, antimicrobial, and anticoagulant resistance. One study has identified cytokines present in both obese and coronavirus-infected patients with an increasing prognosis and pathogenesis for COVID-19 [28]. As obesity weakens the immune system by disrupting the equilibrium of T cells [30], the condition grows more serious and causes the adipose tissue to accumulate proinflammatory cells such as macrophages, dendritic cells, cytotoxic T cells, and Th1 cells. Such changes in immune cells and their concentration in adipose tissue are the major cause of COVID-19 severity in obese people [31]. Furthermore, obesity and insulin resistance might result in a chronic inflammatory state that triggers a cytokine storm, thereby increasing susceptibility to COVID-19 infection. Adipokines such as leptin, resistin, retinol-binding protein 4 (RBP4), and visfatin that are secreted by inflamed adipose tissue can cause systematic increases in levels of proinflammatory cytokines such as TNF-*α*, IL-6, and IL-1 [32].

In the present study, we observed that the levels of *α*-carotene, riboflavin, folate, iron, zinc, magnesium, selenium, omega-3, and fiber declined as the E-DII score increased. Moreover, the E-DII score was positively associated with a higher risk of contracting COVID-19 infection. So, these findings demonstrated the role of antioxidant and anti-inflammatory nutrients in ensuring the optimal functioning of the immune system [33]. It is important to note that consumption of these nutrients was near to Recommended Dietary Allowance (RDA) levels, but it was shown that supplementing some micronutrients above RDA might be required to suppress infection and coronavirus [34].

The above-mentioned nutrients have several roles in improving immune function. They promote recovery from prior inflammatory responses by acting as antioxidants or decreasing certain cytokines [34]. They also play a role in phagocytic and killing activities of neutrophils and macrophages, the development and preservation of physical barriers, the synthesis and function of antimicrobial proteins, and even the growth, differentiation, and motility of cells of the innate immune system [34, 35]. They develop acquired immunity in several ways. For example, iron is important in both health and infection due to its role in epithelial tissue differentiation and growth [36], as well as neutrophils that need iron to produce reactive oxygen species (ROS) to kill pathogens [35].

Zinc is vital for the growth, differentiation, and activation of T lymphocytes [35] and an increase in the intracellular zinc concentration can effectively impair the replication of many ribonucleic acid (RNA) viruses, including coronaviruses [37]. Selenium exerts antioxidant activity through its role in glutathione, thus boosting immunity by reducing inflammation, particularly through protecting neutrophils from oxidative stress by the promotion of glutamine peroxidase [38]. Also, omega-3 fatty acids are powerful mediators of inflammation and thrombosis [39]. Omega-3 fatty acids improve B cells activity, increase phagocytosis, and decrease cytokines as well as inflammatory eicosanoids [40]. In contrast, Omega-6 fatty acids appear to have proinflammatory and prothrombotic characteristics. The high omega-6/omega-3 ratio is extremely proinflammatory, leading to increased expression of CRP, leukotrienes, and ultimately high levels of cytokines including IL-6 of TNF-*α* that could increase the susceptibility of COVID-19 [41]. On the other hand, fiber intake reduces the risk of COVID-19; and such an effect is attributed to its anti-inflammatory and immunostimulant properties. The fermentation of dietary fiber by intestinal bacteria results in the production of short-chain fatty acids (SCFAs) that have been known to regulate host metabolism, cell proliferation, and the immune system [42].

This study showed an inverse association between educational level and the risk of COVID-19. Such a finding was similar to a previous report [43] but different from another study [44]. The discrepancy between our study and the study in Nigeria might be due to other risk factors that have greater impacts on the incidence of COVID-19 in Nigeria. It also is possible that poorer populations with low literacy may have fewer foreign visitors and perform fewer diagnostic tests. Moreover, the literacy rate of the poor population in Nigeria was much lower [44] than that of the present study. There is a link between educational level and factors that increases the risk of COVID-19, including unhealthy dietary habits that suppress the immune defense [43]. The association between malnutrition and the inflammatory state has demonstrated that poor nutrition increases the susceptibility of individuals to COVID-19 [22, 45]. Furthermore, education-related health awareness can play an important role in the incidence and severity of COVID-19. Efficient coordination of public health activities is needed to respond appropriately during a pandemic such as COVID-19 that is dependent on people's ability to access and interpret relevant information, while people with insufficient health literacy might be misled more by false information [46].

The present study did not show any association between DII score, body composition, and anthropometric indices, a finding that is similar to that observed in another study [47]; but divergent from other reports [48, 49]. The discrepancy between findings observed in our study and those of others [48, 49] might be related to case and control group selection. In our study, controls were randomly chosen from the same population as the cases used an identical cohort as the source. Several strong points should be considered in evaluating the present study. COVID-19 is a new disease with limited information and this study has been a pioneer to investigate the E-DII/DII association with COVID-19. Another strength can be the selection of the control group from a population with a common origin with the case group, which did not differ significantly in terms of some confounding parameters such as age, sex, ethnicity, working conditions, and even anthropometric parameters. The use of a verified and reproducible FFQ is another strength, which provided a comprehensive assessment of the usual daily food intake. However, there could be some inherent measurement errors in the FFQ, as we have shown previously [50, 51]. Recall bias is a limitation [52]; however, due to the nature of the study design, recall bias could not be ascribed to the presence or absence of disease, as trained staff administered the FFQ before any infection of the participants. Another potential weakness is the assessment of lack of physical activity, which should be considered in future researches as an important variable when evaluating inflammatory status. Also, the limited number of participants in the case group was another weakness of the study. Thus, to optimize the power of the study, approximately 2.5 controls were included for each case.

## 5. Conclusion

In conclusion, people consuming a more proinflammatory diet and those who were obese were more susceptible to coronavirus infection. Thus, encouraging individuals to maintain their ideal body weight and consume a diet with more anti-inflammatory elements may help them to reduce the probability of acquiring COVID-19 disease.

## Figures and Tables

**Table 1:** Characteristics of participants, COVID-19 case-control study nested within the Cohort Study of Shiraz University of Medical Sciences (SUMS), 2017–2021.

Characteristics	Cases (*n* = 133)	Controls (*n* = 322)	*P* value
Age, (year), mean (SD)	40.13 (7.02)	40.36 (6.56)	0.44
Weight (kg), mean (SD)	74.30 (14.62)	73.46 (13.69)	0.56
Height (cm), mean (SD)	164.74 (8.22)	165.64 (9.24)	0.39
BMI (kg/m^2^), mean (SD)	27.27 (4.34)	26.72 (4.11)	0.23
FFM, kg, mean (SD)	47.96 (10.41)	48.15 (10.95)	0.98
SMM, kg, mean (SD)	26.53 (6.20)	26.62 (6.59)	0.95
BFM, kg, mean (SD)	25.79 (9.44)	25.01 (8.14)	0.52
Circumference of abdomen, cm, mean (SD)	94.32 (12.29)	93.66 (10.91)	0.60
Circumference of hip, cm, mean (SD)	100.10 (7.01)	99.51 (6.59)	0.44
WHR, mean (SD)	0.93 (0.06)	0.93 (0.05)	0.73
VFA, mean (SD)	125.09 (49.89)	121.11 (43.50)	0.54
Gender, N (%)			0.28
Female	78 (58.6)	171 (53.1)	
Male	55 (41.4)	151 (46.9)	
Marital status, N (%)			0.62
Yes	112 (84.2)	265 (82.3)	
No	21 (15.8)	57 (17.7)	
Smoking habits, N (%)			0.45
Nonsmoker	116 (87.2)	272 (84.5)	
Smoker	17 (12.8)	50 (15.5)	
Educational level, N (%)			0.001
Under diploma and diploma degree	7 (5.3)	46 (14.3)	
Postdiploma and bachelor's degree	28 (21.1)	91 (28.3)	
Master's and Ph.D. degree	98 (73.7)	185 (57.5)	
Ethnicity, N (%)			0.14
Fars	106 (79.7)	262 (81.4)	
Lor	9 (6.8)	20 (6.2)	
Turk	9 (6.8)	30 (9.3)	
Others	9 (6.8)	10 (3.1)	

Independent sample *t*-test and independent Mann–Whitney *U* test were used for continuous variables, Chi-square or Fisher's exact test were used for categorical variables. SD: standard deviation; N: number; kg: kilograms; BMI: body mass index; FFM: fat-free mass; SMM: skeletal muscle mass; BFM: body fat mass; WHR: waist-to-hip ratio; VFA: visceral fat area.

**Table 2:** Characteristics of participants according to quartile of E-DII, COVID-19 case-control study nested within the Cohort Study of Shiraz University of Medical Sciences (SUMS), 2017–2021.

Variables	1^st^ quartile^∗^	2^nd^ quartile^∗∗^	3^rd^ quartile^∗∗∗^	4^th^ quartile^∗∗∗∗^	*P* value
Gender, N (%)					0.001
Female	78 (68.4)	61 (53.5)	52 (45.6)	58 (51.3)	
Male	36 (31.6)	53 (46.5)	62 (54.4)	55 (48.7)	
COVID-19, N (%)					≤0.001
Yes	6 (5.3)	18 (15.8)	33 (28.9)	76 (67.3)	
No	108 (94.7)	96 (84.2)	81 (71.1)	37 (32.7)	
Smoking habits, N (%)					0.57
Nonsmoker	100 (87.7)	97 (85.1)	93 (81.6)	98 (80.7)	
Smoker	14 (12.3)	17 (14.9)	21 (18.4)	15 (13.3)	
Age, (year), mean (SD)	41.54 (6.72)	40.75 (6.31)	39.68 (6.32)	39.97 (6.99)	0.16
Weight, (kg), mean (SD)	71.91 (12.88)	73.95 (14.94)	75.94 (13.95)	73.02 (13.85)	0.07
BMI, (kg/m^2^), mean (SD)	26.84 (4.14)	26.94 (4.11)	27.12 (4.28)	26.61 (4.22)	0.74
Food parameters, mean (SD)					
Vitamin A (*μ*g/day)	758.38 (828.96)	792.26 (551.93)	1013.27 (981.08)	1062.02 (615.96)	≤0.001
Βeta-carotene (*μ*g/day)	4195.05 (3751.54)	3623.80 (3427.30)	3346.71 (3190.90)	1665.86 (1695.02)	≤0.001
Vitamin D (*μ*g/day)	1.61 (1.16)	1.65 (1.35)	1.52 (1.88)	1.43 (1.65)	0.01
Vitamin E (mg/day)	22.49 (11.18)	23.86 (14.01)	23.30 (13.80)	26.34 (16.71)	0.28
Vitamin C (mg/day)	184.45 (112.36)	196.3 (135.82)	177.58 (99.82)	181.61 (90.06)	0.94
Thiamin, B1 (mg/day)	2.67 (2.13)	2.52 (1.06)	2.41 (1.16)	2.20 (0.65)	0.23
Riboflavin, B2 (mg/day)	2.75 (1.75)	2.49 (1.11)	2.36 (1.27)	2.08 (0.70)	0.001
Niacin, B3 (mg/day)	28.95 (20.71)	28.18 (10.45)	26.96 (11.56)	25.61 (7.36)	0.37
Pyridoxine, B6 (mg/day)	2.36 (1.52)	2.36 (0.85)	2.28 (0.93)	2.17 (0.56)	0.64
Folate, B9 (*μ*g/day)	734.36 (383.95)	719.10 (293.84)	640.19 (306.29)	498.97 (204.57)	≤0.001
Cobalamin, B12 (*μ*g/day)	4.13 (7.56)	5.87 (2.46)	4.01 (4.37)	4.30 (3.17)	0.28
MUFA (g/day)	29.13 (14.50)	28.83 (11.77)	26.12 (11.31)	26.19 (11.59)	0.10
PUFA (g/day)	22.96 (10.06)	23.59 (10.96)	22.62 (11.03)	27.29 (13.45)	0.01
Omega-6 (g/day)	20.52 (9.37)	21.15 (10.53)	20.77 (10.62)	24.41 (13.01)	0.02
Omega-3 (g/day)	1.03 (0.71)	1.00 (0.68)	0.81 (0.50)	0.65 (0.46)	≤0.001
Iron (mg/day)	43.16 (42.28)	39.77 (32.85)	36.93 (32.51)	25.78 (13.07)	≤0.001
Zinc (mg/day)	14.91 (10.22)	13.27 (4.71)	12.53 (4.91)	11.64 (3.61)	0.002
Magnesium (mg/day)	512.65 (428.63)	452.20 (170.55)	423.79 (187.68)	362.69 (120.01)	≤0.001
Selenium (mg/day)	134.30 (175.36)	109.93 (69.03)	89.27 (82.70)	40.29 (63.13)	≤0.001
Fiber (g/day)	59.15 (46.81)	60.99 (41.85)	47.72 (26.17)	31.55 (18.21)	≤0.001
Total fat (g/day)	79.49 (39.98)	79.69 (28.67)	74.44 (28.31)	82.10 (29.33)	0.23
Cholesterol (mg/day)	245.29 (154.93)	261.50 (137.20)	240.39 (177.10)	263.85 (116.31)	0.03
Saturated fat (g/day)	19.05 (15.30)	19.07 (6.99)	17.82 (7.12)	18.97 (6.13)	0.33
Trans-fat (g/day)	00 (00)	00 (00)	00 (00)	00 (00)	0.43

^∗^Quartile 1 = ≤−2.76;  ^*∗*^ ^*∗*^Quartile 2 = −2.75 – (-) 1.65;  ^*∗*^ ^*∗*^ ^*∗*^Quartile 3 = −1.64 – (-) 0.55;  ^*∗*^ ^*∗*^ ^*∗*^ ^*∗*^Quartile 4 ≥ −0.54. ANOVA or Kruskal–Wallis test was used for continuous variables and Chi-square was used for categorical variables. E-DII: energy-adjusted dietary inflammatory index; BMI: body mass index; BFM: body fat mass; WHR: waist-to-hip ratio; FFM: fat-free mass; SMM: skeletal muscle mass; VFA: visceral fat area.

**Table 3:** Univariate and multivariate logistic model for odds of COVID-19 infection, COVID-19 case-control study nested within the Cohort Study of Shiraz University of Medical Sciences (SUMS), 2017–2021.

Variables	Univariate model				Multivariate model			
	OR	95% CI		*P* value	OR	95% CI		*P* value
		Lower	Upper			Lower	Upper	
E-DII	2.67	2.18	3.27	≤0.001	2.86	2.30	3.55	≤0.001
Age	0.98	0.95	1.01	0.46				
Weight	1.00	0.99	1.01	0.55				
WHR	1.09	0.04	29.79	0.95				
BFM	1.01	0.98	1.03	0.37				
BMI	1.03	0.98	1.08	0.20	1.07	1.01	1.13	0.02
Gender								
Male	Ref							
Female	0.79	0.53	1.20	0.28				
Educational level								
Under diploma and diploma degree	Ref				—	—	—	—
Postdiploma and bachelor's degree	0.28	0.12	0.66	0.003	0.15	0.05	0.40	≤0.001
Master's and PhD degree	0.58	0.35	0.94	0.03	0.52	0.29	0.92	0.02
Smoking habits								
No	Ref							
Yes	1.25	0.69	2.26	0.45				

Univariate and multivariate logistic models were used. OR: odds ratio; CI: confidence interval; Ref: reference; E-DII: energy-adjusted dietary inflammatory index; WHR: waist-to-hip circumference rate; BFM: body fat mass; BMI: body mass index.

## Data Availability

The data used to support the findings of this study are available from the corresponding author upon request.

## List of Abbreviations

BFM: Body fat mass
BMI: Body mass index
CI: Confidence intervals
COVID-19: SARs CoV2 coronavirus disease
CRP: C-reactive protein
DII: Dietary inflammatory index
E-DII: Energy-adjusted dietary inflammatory index
FEV1: Forced expiratory volume1
FFM: Fat-free mass
FFQ: Food frequency questionnaire
IL-6: Interleukin-6
OR: Odds ratio
qPCR: Quantitative real-time polymerase chain reaction
RBP4: Retinol-binding protein 4
RDA: Recommended Dietary Allowance
RNA: Ribonucleic acid
ROS: Reactive oxygen species
SCFAs: Short-chain fatty acids
SMM: Skeletal muscle mass
SUMS: Shiraz university of medical sciences
TNF-*α*: Tumor necrosis factor alpha
USDA: United States department of agriculture
VCAM: Vascular cell adhesion molecules
VFA: Visceral fat area
WHO: World Health Organization
WHR: Waist-to-hip ratio.

## References

[1] Jin Y., Yang H., Ji W. (2020). Virology, epidemiology, pathogenesis, and control of COVID-19. *Viruses*.

[2] WHO Novel coronavirus (2019-nCoV) situation reports world health organization (WHO) 2021. https://www.who.int/emergencies/diseases/novel-coronavirus-2019/situation-reports/.

[3] Nizami N. S., Mujeebuddin C. (2020). Strong immunity-a major weapon to fight against Covid-19. *IOSR Journal of Pharmacy and Biological Science*.

[4] Asif M., Saleem M., Saadullah M., Yaseen H. S., Al Zarzour R. (2020). COVID-19 and therapy with essential oils having antiviral, anti-inflammatory, and immunomodulatory properties. *Inflammopharmacology*.

[5] Moludi J., Alizadeh M., Jafari Vayghan H., Naemi M., Rahimi A., Musavi R. (2021). The relationship between dietary inflammatory index (DII) and disease severity and inflammatory status: a case-control study of COVID-19 patients. *British Journal of Nutrition*.

[6] Maffetone P. B., Laursen P. B. (2020). The perfect storm: coronavirus (Covid-19) pandemic meets overfat pandemic. *Frontiers in Public Health*.

[7] Coperchini F., Chiovato L., Croce L., Magri F., Rotondi M. (2020). The cytokine storm in COVID-19: an overview of the involvement of the chemokine/chemokine-receptor system. *Cytokine & Growth Factor Reviews*.

[8] BourBour F., Mirzaei Dahka S., Gholamalizadeh M. (2020). Nutrients in prevention, treatment, and management of viral infections; special focus on coronavirus. *Archives of Physiology and Biochemistry*.

[9] Iddir M., Brito A., Dingeo G. (2020). Strengthening the immune system and reducing inflammation and oxidative stress through diet and nutrition: considerations during the COVID-19 crisis. *Nutrients*.

[10] Barbaresko J., Koch M., Schulze M. B., Nöthlings U. (2013). Dietary pattern analysis and biomarkers of low-grade inflammation: a systematic literature review. *Nutrition Reviews*.

[11] Giugliano D., Ceriello A., Esposito K. (2006). The effects of diet on inflammation. *Journal of the American College of Cardiology*.

[12] Wirth M. D., Shivappa N., Davis L. (2017). Construct validation of the dietary inflammatory index among African Americans. *The Journal of Nutrition, Health & Aging*.

[13] Han Y. Y., Forno E., Shivappa N., Wirth M. D., Hébert J. R., Celedón J. C. (2018). The dietary inflammatory index and current wheeze among children and adults in the United States. *Journal of Allergy and Clinical Immunology: In Practice*.

[14] Sideleva O., Suratt B. T., Black K. E. (2012). Obesity and asthma: an inflammatory disease of adipose tissue not the airway. *American Journal of Respiratory Critical Care Medicine*.

[15] Malekshah A. F., Kimiagar M., Saadatian-Elahi M. (2006). Validity and reliability of a new food frequency questionnaire compared to 24 h recalls and biochemical measurements: pilot phase of golestan cohort study of esophageal cancer. *European Journal of Clinical Nutrition*.

[16] Patry-Parisien J., Shields M., Bryan S. (2012). Comparison of waist circumference using the world health organization and national institutes of health protocols. *Health Reports*.

[17] Shivappa N., Steck S. E., Hurley T. G., Hussey J. R., Hébert J. R. (2014). Designing and developing a literature-derived, population-based dietary inflammatory index. *Public Health Nutrition*.

[18] Hébert J. R., Shivappa N., Wirth M. D., Hussey J. R., Hurley T. G. (2019). Perspective: the dietary inflammatory index (DII)—lessons learned, improvements made, and future directions. *Advances Nutrition*.

[19] Wood L. G., Shivappa N., Berthon B. S., Gibson P. G., Hebert J. R. (2015). Dietary inflammatory index is related to asthma risk, lung function and systemic inflammation in asthma. *Clinical & Experimental Allergy*.

[20] Fowler M. E., Akinyemiju T. F. (2017). Meta‐analysis of the association between dietary inflammatory index (DII) and cancer outcomes. *International Journal of Cancer*.

[21] Phillips C. M., Chen L.-W., Heude B. (2019). Dietary Inflammatory index and non-communicable disease risk: a narrative review. *Nutrients*.

[22] Mackay C. R. (2020). *The Autoimmune Diseases*.

[23] Wood L. G., Attia J., McElduff P., McEvoy M., Gibson P. G. (2010). Assessment of dietary fat intake and innate immune activation as risk factors for impaired lung function. *European Journal of Clinical Nutrition*.

[24] Wood L. G., Garg M. L., Smart J. M., Scott H. A., Barker D., Gibson P. G. (2012). Manipulating antioxidant intake in asthma: a randomized controlled trial. *The American Journal of Clinical Nutrition*.

[25] Zhou Y., Fu B., Zheng X. (2020). Pathogenic T-cells and inflammatory monocytes incite inflammatory storms in severe COVID-19 patients. *National Science Review*.

[26] Takemura M., Matsumoto H., Niimi A. (2006). High sensitivity C-reactive protein in asthma. *European Respiratory Journal*.

[27] Cai Q., Chen F., Wang T. (2020). Obesity and COVID-19 severity in a designated hospital in Shenzhen, China. *Diabetes Care*.

[28] Petrakis D., Margină D., Tsarouhas K. (2020). Obesity-a risk factor for increased COVID‑19 prevalence, severity and lethality (review). *Molecular Medicine Reports*.

[29] Korakas E., Ikonomidis I., Kousathana F. (2020). Obesity and COVID-19: immune and metabolic derangement as a possible link to adverse clinical outcomes. *American Journal of Physiology-Endocrinology and Metabolism*.

[30] Misumi I., Starmer J., Uchimura T., Beck M. A., Magnuson T., Whitmire J. K. (2019). Obesity expands a distinct population of T cells in adipose tissue and increases vulnerability to infection. *Cell Reports*.

[31] Popkin B. M., Du S., Green W. D. (2020). Individuals with obesity and COVID‐19: a global perspective on the epidemiology and biological relationships. *Obesity Reviews*.

[32] Vepa A., Bae J. P., Ahmed F., Pareek M., Khunti K. (2020). COVID-19 and ethnicity: a novel pathophysiological role for inflammation. *Diabetes & Metabolic Syndrome: Clinical Research & Reviews*.

[33] Pansarasa O., Pistono C., Davin A. (2019). Altered immune system in frailty: genetics and diet may influence inflammation. *Ageing Research Reviews*.

[34] Calder P., Carr A., Gombart A., Eggersdorfer M. (2020). Optimal nutritional status for a well-functioning immune system is an important factor to protect against viral infections. *Nutrients*.

[35] Gombart A. F., Pierre A., Maggini S. (2020). A review of micronutrients and the immune system-working in harmony to reduce the risk of infection. *Nutrients*.

[36] Núñez G., Sakamoto K., Soares M. P. (2018). Innate nutritional immunity. *Journal of Immunology (Baltimore, Md.: 1950)*.

[37] Te Velthuis A. J. W., van den Worm S. H. E., Sims A. C., Baric R. S., Snijder E. J., van Hemert M. J. (2010). Zn2+ inhibits coronavirus and arterivirus RNA polymerase activity in vitro and zinc ionophores block the replication of these viruses in cell culture. *PLoS Pathogens*.

[38] Avery J., Hoffmann P. (2018). Selenium, selenoproteins, and immunity. *Nutrients*.

[39] Hathaway D., Pandav K., Patel M. (2020). Omega 3 fatty acids and COVID-19: a comprehensive review. *Infection & Chemotherapy*.

[40] Gutiérrez S., Svahn S. L., Johansson M. E. (2019). Effects of omega-3 fatty acids on immune cells. *International Journal of Molecular Science*.

[41] Kain V., Ingle K. A., Kachman M. (2018). Excess *ω*-6 fatty acids influx in aging drives metabolic dysregulation, electrocardiographic alterations, and low-grade chronic inflammation. *American Journal of Physiology-Heart and Circulatory Physiology*.

[42] Conte L., Toraldo D. M. (2020). Targeting the gut–lung microbiota axis by means of a high-fibre diet and probiotics may have anti-inflammatory effects in COVID-19 infection. *Therapeutic Advances in Respiratory Diseases*.

[43] Wall C. L., Gearry R. B., Pearson J., Parnell W., Skidmore P. M. (2014). Dietary intake in midlife and associations with standard of living, education and nutrition literacy. *The New Zealand Medical Journal*.

[44] Hassan Z., Hashim M. J., Khan G. (2020). Population risk factors for COVID-19 deaths in Nigeria at sub-national level. *The Pan African Medical Journal*.

[45] Butler M. J., Barrientos R. M. (2020). The impact of nutrition on COVID-19 susceptibility and long-term consequences. *Brain, Behavior, and Immunity*.

[46] Berkman N. D., Sheridan S. L., Donahue K. E., Halpern D. J., Crotty K. (2011). Low health literacy and health outcomes: an updated systematic review. *Annals of Internal Medicine*.

[47] Haji-Hosseini-Gazestani N., Keshavarz S. A., Hosseini-Esfahani F., Ataie-Jafari A. (2020). The association of dietary inflammatory index and obesity phenotypes in women. *Food & Health.*.

[48] Vahid F., Bourbour F., Gholamalizadeh M. (2020). A pro-inflammatory diet increases the likelihood of obesity and overweight in adolescent boys: a case–control study. *Diabetology Metabolic Syndrome*.

[49] Ruiz-Canela M., Zazpe I., Shivappa N. (2015). Dietary inflammatory index and anthropometric measures of obesity in a population sample at high cardiovascular risk from the PREDIMED (PREvención con DIeta MEDiterránea) trial. *British Journal of Nutrition*.

[50] Hebert J. R., Ma Y., Clemow L. (1997). Gender differences in social desirability and social approval bias in dietary self-report. *American Journal of Epidemiology*.

[51] Hebert J. R., Hurley T. G., Peterson K. E. (2008). Social desirability trait influences on self-reported dietary measures among diverse participants in a multicenter multiple risk factor trial. *The Journal of Nutrition*.

[52] Schlesselman J. J. (1668). *Case-Control Studies: Design, Conduct, Analysis*.

